# Comparative efficacy of a hydroxyapatite and a fluoride toothpaste for prevention and remineralization of dental caries in children

**DOI:** 10.1038/s41405-019-0026-8

**Published:** 2019-12-09

**Authors:** Bennett T. Amaechi, Parveez Ahamed AbdulAzees, Dina Ossama Alshareif, Marina Adel Shehata, Patrícia Paula de Carvalho Sampaio Lima, Azadeh Abdollahi, Parisa Samadi Kalkhorani, Veronica Evans

**Affiliations:** Department of Comprehensive Dentistry, School of Dentistry, University of Texas Health San Antonio, 7703 Floyd Curl Drive, San Antonio, TX 78229-3900 USA

**Keywords:** Oral diseases, Paediatric dentistry

## Abstract

**Objective:**

This in situ study compared the effectiveness of two toothpastes containing hydroxyapatite or 500 ppm fluoride in promoting remineralization and inhibiting caries development.

**Materials and methods:**

Two enamel blocks (human primary teeth), one sound and one with artificially-produced caries lesion, were exposed to toothpaste containing either 10% hydroxyapatite or 500 ppm F^−^ (amine fluoride) via intra-oral appliance worn by 30 adults in two-arm double blind randomized crossover study lasting 14 days per arm (ClinicalTrials.gov: NCT03681340). Baseline and post-test mineral loss and lesion depth (LD) were quantified using microradiography. One-sided *t*-test of one group mean was used for intragroup comparison (baseline vs. post-test), while two-sided *t*-test of two independent means was used to compare the two toothpaste groups.

**Results:**

Pairwise comparison (baseline vs. test) indicated significant (*p* < 0.0001) remineralization and LD reduction by either toothpaste; however, when compared against each other, there was no statistically significant difference in remineralization or LD reduction between the two toothpastes. No demineralization could be observed in sound enamel blocks exposed to either toothpaste. While F^−^ induced lesion surface lamination, HAP produced a more homogenous lesion remineralization.

**Conclusions:**

10% hydroxyapatite achieved comparable efficacy with 500 ppm F^−^ in remineralizing initial caries and preventing demineralization. Thus the HAP toothpaste is confirmed to be equal to the fluoride toothpaste in this study.

## Introduction

Although preventable, dental caries continues to be one of the most prevalent chronic diseases among children in the U.S. and the world, and one of the most common unmet healthcare needs of poor children.^1^ As much as 80% of caries incidence is experienced by only 20–25% of children, with 10% having untreated cavities, and those from low socioeconomic and minority groups experiencing significantly higher rates and at younger ages.^2,3^

It is well documented that saliva has a caries protective effects due to its supersaturation with Ca^2+^ and PO_4_^3–^ ions in a bioavailable form and enrichment with various proteins playing multiple roles in maintenance of hard tissue integrity throughout life.^4^ Furthermore, the supersaturation of saliva with Ca^2+^ and PO_4_^3–^ ions, at physiological pH, ensures that these ions are bioavailable to diffuse into mineral deficient lesions to induce remineralization.^5^ However, the natural caries protective and remineralizing effects of saliva is not only a slow process but obviously insufficient to protect individuals against caries and remineralize existing lesions without additional agents to enhance its effects.

Although fluoride interventions seem to have the most consistent benefit in preventing caries development and remineralizing initial lesions with the highest level of supporting evidence,^6–8^ caries still develop in high risk individuals of all ages, irrespective of the dose of fluoride used.^9,10^ There are limitations to what application of fluoride alone can achieve in relation to caries prevention and remineralization.^11^ These limitations may be associated with facts that fluoride becomes less effective below a pH of about 4.5;^12^ fluoride still needs Ca^2+^ and PO_4_^3–^ ions in a bioavailable form in saliva and other sources to be effective; and fluoride remineralization of initial lesions is most effective at the outer 30 μm of the lesion,^13,14^ thus leading to surface-zone remineralization at the expense of the lesion body, making full remineralization difficult to achieve.^15,16^ Furthermore, although the efficacy of fluoride is dose-dependent and increases with increase dose,^7^ there is a limit to which you can increase the dosage of fluoride to avoid the risk of fluorosis in children^17^ and toxicity in all ages.^18,19^ The effect of dose limitation on fluoride effectiveness may be more pronounced in children below 6 years, since the fluoride dose recommended for this group is even lower than the regulatory 1000–1500 ppm fluoride concentration in non-prescription toothpastes, and as such probably suboptimal for effective remineralization of initial lesions.

The above mentioned limitations of the saliva homeostatic mechanisms and fluoride-based strategies in caries prevention and remineralization, especially in highly cariogenic oral environments justify the need for new-age strategies that could work either better than or as effective as fluoride but can permit increasing dosage for increase effectiveness without safety concerns. It is envisaged that the presence of additional extrinsic sources of stabilized Ca^2+^ and PO_4_^3–^ ions could augment the natural caries preventive and remineralization potential of saliva by increasing diffusion gradients favoring faster and deeper subsurface remineralization.^20^ One of the new caries remineralizing technologies is the biomimetic systems, among which are the synthetic hydroxyapatite (HAP; Ca_5_(PO_4_)_3_(OH) applied in microcluster or nanocrystalline forms in oral care products.^21,22^ HAP is a bioactive and biocompatible material with similar chemical composition to the apatite crystals of human enamel.^21–24^ Several in vitro and in situ studies have provided evidences supporting the caries remineralization and prevention potential of HAP in oral care products based on its demonstrated ability to strongly adsorb to tooth surfaces, plaque components and bacteria.^24–29^ Randomized controlled clinical trials, some of which have led to the approval of HAP as an anti-caries agent in Japan in 1993 and in Canada in 2015, have demonstrated its non-inferiority and equivalence to fluoride.^30–32^ Therefore, the objective of this in situ study was to determine whether Karex Kid’s toothpaste containing 10% HAP microclusters (Kinder Karex Zahnpasta, Dr. Kurt Wolff GmbH and Co. KG, Bielefeld, Germany) is as effective as Elmex Kid’s toothpaste containing 500 ppm fluoride as amine fluoride (Elmex Kinder Zahnpasta, CP GABA GmbH, Hamburg, Germany) in promoting the remineralization and inhibiting the development of carious lesions. We hypothesized that (1) each of the two toothpaste formulations promotes remineralization and lesion depth reduction that is significantly greater than zero, and (2) neither toothpaste is inferior to the other with respect to promoting the remineralization and inhibiting the development of carious lesions.

## Materials and methods

This is a double-blind, randomized, crossover, single center, controlled in situ study to establish the equivalence of two children toothpaste formulations, containing either 10% HAP microclusters (crystallite size: length ≈ 80 nm (median) × width ≈ 30 nm (median)) or 500 ppm fluoride provided as amine fluoride (AMF), in inducing the remineralization and inhibiting the development of initial caries lesions. The primary outcomes to be examined were (1) the percentage remineralization and lesion depth reduction measured relative to the baseline mineral loss and lesion depth for initial caries, and (2) the amount of mineral loss and lesion depth for the sound enamel. The study was conducted at the clinical research facility of the University of Texas Health San Antonio (UTHSA) School of Dentistry. The UTHSA Institutional Review Board (IRB) approved the study (protocol #: HSC20180416H), and the study was registered with ClinicalTrials.gov (NCT03681340). The study was conducted in accordance with the ethical standards outlined in the 1964 Declaration of Helsinki and its later amendments, and in compliance with the International Conference on Harmonization (ICH) Good Clinical Practice Guidelines. The participants were recruited from among different ethnic origins and varied socio-economic status in the local San Antonio area, written informed consent was obtained from all participants prior to their participation in the study.

### Participant recruitment

**Fig. 1:**
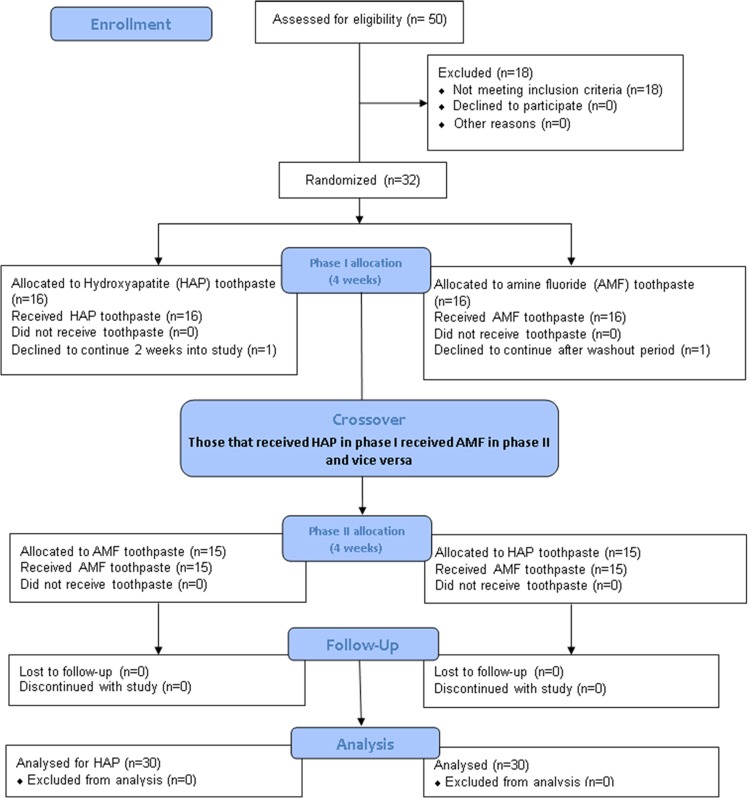
Flow Diagram detailing the stepwise methodology. This is a crossover study so the 30 completers received the two intervention in a crossover design as phase I and II.

Fifty subjects aged from 18 to 60 years were given screening examination that included sialometry, medical/dental history, and oral examination (Fig. 1). Thirty two subjects qualified and were enrolled in the study. Inclusion criteria were age of 18 through 60 years; normal salivary function with unstimulated and stimulated salivary flow rates ≥0.2 ml/min and ≥0.7 ml/min, respectively, measured according to the Sreebny and Valdini^33^ procedure. Other inclusion criteria were not taking any antibiotics or medications which could affect saliva flow rate; the presence of at least 20 natural uncrowned teeth (excluding third molars); a past history of dental caries but no clinically active caries; willing to wear the in situ appliance and use only assigned products for oral hygiene throughout the duration of the study; the ability to read and understand English; ability to provide informed consent; and no self-reported history of allergy to personal care/consumer products or any ingredient in the test products. Exclusion criteria were the presence of advanced periodontal disease or other oral pathology; medical condition that requires premedication prior to dental procedures; use of antibiotics one month prior to or during this study; self-reported pregnancy or breastfeeding; and use of tobacco products.

### Creation of artificial initial caries and fabrication of the in situ appliance

Following consent from the donors, freshly extracted primary teeth were collected from the pediatric clinics of the UTHSA School of dentistry and stored in 0.1% thymol solution at 4 °C prior to use. The teeth were examined with a transilluminator, and thirty two teeth without caries, cracks, or enamel malformations were selected and cleaned with pumice using electronic toothbrush. Using a water-cooled diamond wire saw, 4 tooth blocks were produced from buccal and lingual surfaces of each of the selected teeth, with each block measuring ~2 mm length × 2 mm width × 1.5 mm thickness. Two of the 4 blocks were retained as sound enamel blocks for demineralization inhibition assessment while the other two blocks targeted for remineralization assessment had artificial initial caries produced in them as follows. All surfaces of each block were painted with two coats of acid resistant nail varnish except buccal or lingual on which an initial caries lesion was created by subjecting this exposed surface to 7 days demineralization in an acidified gel system (0.10 M lactic acid, 0.10 M sodium hydroxide, 6% ^w^/_v_ hydroxyethyl cellulose, pH 4.5). Following lesion formation, the nail varnish was carefully removed with acetone. A tooth section (~150 µm thick) was cut from each tooth block for the measurement of the baseline mineral loss (∆z_1_) and lesion depth (LD_1_) of each produced initial caries lesion, and for selection of the suitable lesions for the remineralization assessment. The sections were processed for transverse microradiography (TMR) as follows. Both sides of the sections were polished using adhesive back lapping film in a MultiPrep™ Precision Polishing machine (Allied High Tech, USA) to achieve planoparallel surfaces, as well as reduce the thickness of the slice to 100 µm (the appropriate thickness for TMR). Following this, the sections were microradiographed on a type lA high resolution glass X-ray plate (Microchrome Technology, CA, USA) using a Phillips X-ray generator system set up for this purpose. The plates were exposed for 10 min at an anode voltage of 20 kV and a tube current of 10 mA, and then processed. Processing consisted of a 5 min development in Kodak HR developer and 15 min fixation in Kodak Rapid-fixer before a final 30 min wash period. After drying, the microradiographs were examined under a Leica DMR optical microscope linked via a Sony model XC-75CE CCTV camera to a personal computer. Using TMR2006 version 3.0.0.11 image analysis software (Inspektor Research Systems, Amsterdam, Netherlands), the enhanced image of the microradiographs were analyzed under standard conditions of light intensity and magnification along with the image of a step wedge as described by de Josselin de Jong et al.^34^ At this point, the images were used only for selection of the suitable lesions for the study. Only the controls that showed caries-like lesions with subsurface lesions, which display a fairly uniform width throughout their length, were selected for the remineralization process, and their test blocks were used for construction of the in situ appliance. It is pertinent to mention that the baseline measurements were not conducted for the sound tooth blocks to be used for demineralization-prevention study because the TMR does not measure the mineral density of sound tooth tissue rather the software uses the known mineral density of sound enamel or dentin to determine the amount of mineral loss in a demineralized tissue.

As stated above, the four tooth blocks from each tooth were distributed as follows**:** two lesion-bearing blocks for remineralization assessment and two sound blocks for demineralization inhibition assessment. These four blocks were used to fabricate the in situ appliances as follows. Each block was covered with polyester gauze (Bard Peripheral Vascular, Inc., Tempe, AZ, USA) and mounted within an in situ appliance, a customized orthodontic bracket.^27^ The polyester gauze facilitated plaque retention on the surface of the tooth blocks on intra-oral exposure. The appliance consists of an orthodontic molar pad with retentive mesh backing (American Orthodontics Corp., Sheboygan, US), which has a ring of 0.7 mm orthodontic wire welded to it so that the ring closely encircles each test-block. The block was retained within the bracket with fluoride-free intermediate restorative material (IRM). All appliances were sterilized with gamma irradiation prior to delivery to the subject.

### Study treatment

The study was performed in two distinct treatment phases during which subjects were exposed to one of the following two treatments in a randomized crossover design; (A) Karex Kid’s toothpaste containing 10% HAP microclusters (Kinder Karex, Dr. Kurt Wolff GmbH and Co. KG, Bielefeld, Germany), and (B) Elmex Kid’s toothpaste containing 500 ppm fluoride as AMF (Elmex Kinder zahnpasta, GABA GmbH, Hamburg, Germany). Each phase started with one week of washout period and then 4 weeks of treatment, consisting of two 2-week periods during which each subject used his/her assigned treatment under the following conditions. First 2-week period, with the subject wearing an in situ appliance with sound enamel block, and the second 2-week period, with the subject wearing an in situ appliance with lesion-bearing tooth block.

Subjects who satisfied enrollment criteria were given a specially manufactured washout toothpaste with neither fluoride nor HAP (Dr. Kurt Wolff GmbH and Co. KG, Bielefeld, Germany) and an adult soft-bristled toothbrush to use for a washout period of one week. The washout period allows for attenuation of any residual effect of the subject's previously used toothpaste. There was no washout period between individual 2-week treatment periods within each phase since the subjects used the same product for the 4 weeks. During the washout period, subjects were instructed to use the toothpaste and toothbrush for 2 min twice a day (morning after breakfast and night last thing before bed) in place of their normally used toothpaste and toothbrush, and as their only oral hygiene product. Subjects were given no restriction on dietary habit.

At the end of the washout period, patients returned to the clinic, and were assigned to a group to use either HAP or AMF by the Study Coordinator using randomization numbers generated by a computer program designed and operated by our biostatistics team. However, to ensure that both the operators and the subjects were blinded as to product assignment, all toothpaste tubes were packaged identically and coded (A, B, or washout) by the manufacturing/packaging company, who retained the code until the completion of the study and data interpretation. Following randomization, the 4 block-bearing in situ appliances originating from one tooth were assigned to one subject. Then the first of the four assigned appliances was bonded, in accordance with current principles of orthodontic practice, on the buccal surface of the chosen lower molar tooth. The appliance was bonded by a qualified dentist licensed in the state of Texas, who was different from the laboratory technician that process and analyze the samples to produce the final data. To bond the appliance, the buccal surface of the tooth chosen was carefully etched for 30 s, washed with water spray and dried for a further 30 s, and isolated using cotton rolls. The bottom of the appliance was loaded with Transbond™ XT light-cure adhesive paste (3M Unitek, Monrovia, CA, USA), and carefully positioned to avoid causing occlusal interference and to avoid soft tissue irritation. The excess composite material that spilled out from the sides of the appliance was used to cover the sides, beveling it to present a comfortable streamlined (non-catching) surface when the slab comes in contact with a soft tissue surface (e.g., tongue). The adhesive paste was cured using an Ortholux XT visible Light Curing Unit (3M Unitek, Monrovia, Ca, USA) applied for 20 s.

Following bonding of the appliance, each subject was given his/her respective test toothpaste and a soft-bristled toothbrush designed for use with orthodontic brackets with shorter bristles at the center to accommodate the bracket. Subjects were instructed to continue with the routine of brushing two times daily, morning after breakfast and last thing before bed, for 2 min before rinsing with only 10 ml of water. Subjects were also given special instruction on dispensing of the toothpaste, and were advised not to brush directly on the appliance but rather to brush around it to prevent disruption of the dental plaque on the surface of the tooth block. Subjects were restricted from eating nor drinking for at least 30 min after brushing. A timer and measuring cup were provided to each subject. As a method of monitoring compliance, a diary was provided to each subject for recording the time of each brushing episode, and in addition, toothpaste tubes were weighed at the time of randomization and at each study visit. Subjects were instructed to maintain their normal dietary habits and were prohibited from using any other oral hygiene product (e.g., mouthwash, chewing gum) or tooth-whitening product for the duration of the study. Immediately after bonding of the first appliance, each subject used the test product under the supervision of the Study Coordinator, and for the remainder of each treatment phase, subjects completed the procedure at home and as instructed by the Study Coordinator

Subjects returned to the clinical research facility after 2 weeks without using the product that morning, and the first appliance was detached and sent to the laboratory for analysis. The appliance for the second 2-week treatment period of the phase was bonded, the dairy checked, the toothpaste weighed, and safety evaluation performed. Upon completion of the second 2-week treatment period, the subject again returned to the clinic for detachment of the second appliance, and was given washout toothpaste and a soft-bristle toothbrush to undergo another 7-day washout period without an appliance in preparation for his/her phase 2 of the study. After completion of the washout period without an appliance, subjects return to the clinic, and the procedure of phase 1 was repeated until the second 2-week treatment period was completed, and each subject has gone through the two arms of the study.

### Safety monitoring

At all visits, the dental examiner visually examines the oral cavity and peri-oral area, and this examination included an evaluation of the soft and hard palate, gingival mucosa, buccal mucosa, mucogingival fold areas, tongue, sublingual and submandibular areas, salivary glands, and the tonsillar and pharyngeal areas. In addition to oral examination, subjects were screened for adverse events using a questionnaire.

### Post-treatment processing and study outcomes

Following intra-oral exposure, a tooth section (~150 µm thick) was cut from each tooth block, both sound and lesion-bearing blocks. The sections were processed for microradiography as described above for the control sections used for baseline data. Although the lesion-bearing control sections have been microradiographed and analyzed for selection of the appropriate lesions, they were microradiographed again together with the post-test sections and both analyzed together for quantification of the ∆z and LD of the lesions as described for baseline sections. This step enabled both control and test sections from same block to be microradiographed and analyzed under the same conditions. For the lesion-bearing sections, this process yielded the pre-test mineral loss (∆z_1_) and lesion depth (LD_1_), the post-test mineral loss (∆z_2_), and lesion depth (LD_2_), and the pre-test and post-test microradiograms of the lesions. For the sections from sound tooth blocks, the process yielded the post-test mineral loss (∆z) and lesion depth (LD) if any lesion developed, and the microradiograms. Using the microradiograms, the pattern and the extent of remineralization produced within each lesion by each treatment arm was examined by comparing the pre-test and post-test images side-by-side. For each participant the post-treatment mineral loss was subtracted from the pre-treatment mineral loss, and then standardized across participants by dividing that difference by the pre-treatment mineral loss to obtain the % remineralization. The lesion depth pre-treatment and post-treatment was handled the same way to obtain the % lesion depth reduction. The two products were compared using the % remineralization and the % lesion depth reduction.

### Power analysis and sample size calculation

The sample size calculations, which were based on a power analysis, were performed using nQuery Advisor software (Statistical Solutions, Cork, Ireland). Based on previous studies in which the mean % remineralization was equal to 30.3 with a standard deviation equal to 16.3,^27,35–37^ and for the hypothesis that each of the two toothpaste formulations promotes remineralization and lesion depth reduction that is significantly greater than zero, an effective sample size of 30 subjects will have power greater than 0.95 with a 0.05 one-sided significance level to detect a difference between a hypothesis mean of zero and a sample mean % remineralization equal to or greater than 10% using a two-sided *t*-test of two independent means. However, 32 subjects were enrolled to make provision for 5% dropout.

### Statistical analysis

For measurements by both mineral loss and lesion depth, three endpoints were analyzed. (1) The mean amount of remineralization and mean amount of lesion depth reduction were determined for Karex toothpaste as a percentage of pre-treatment mineral loss and pre-treatment lesion depth respectively, and these percentages were compared to a value of 0%, which is what would be expected if the toothpaste had no effect. The statistical test used was a one-sided *t*-test of one group mean. (2) In the same way, the mean amount of remineralization and mean amount of lesion depth reduction was determined for Elmex toothpaste, and also compared to 0%. (3) Finally, the primary endpoint was to compare the means of Karex toothpaste to Elmex toothpaste to check for non-inferiority/equivalence of the HAP toothpaste to the fluoride toothpaste, using the two-sided *t*-test of two independent means. Non-inferiority/equivalence was established if the difference between the two toothpaste formulations for any one measurement method was not regarded to be clinically relevant and was set to Δ ≤ 20%. The statistical package R, version 3.5.0, was used for analysis.

## Results

Of the 32 subjects recruited into this study, 1 subject declined to participate further during the washout period, and one other subject declined to participate midway into the first 2-week treatment while wearing his first appliance that bears sound tooth block (Fig. 1). Thirty subjects (19 females, 11 males) with a mean (SD) age of 39.5 (15.0) years completed the study. The unstimulated and stimulated saliva flow rates of the subjects ranged from 0.2 to 1.5 ml/min and 0.9 to 3.5 ml/min respectively. The ethnic distribution of the subjects was as follows: Hispanic 17 (57%), Black (not Hispanic) 3 (10%), White (not Hispanic) 7 (23%), Asian 1 (3%), and others 2 (7%).

The mean rates of remineralization and lesion depth reduction are shown in Tables 1 and 2. Each toothpaste had a mean percent remineralization in excess of 50%, and a mean percent lesion depth reduction better than 25%. For both toothpastes, the mean % remineralization and mean % lesion depth reduction were statistically significantly greater than 0% (*p* < 0.0001). When compared against each other, there was no statistically significant difference in remineralization (*p* = 0.81), or in lesion depth reduction (*p* = 0.68). The 95% confidence interval of the difference between Karex™ (HAP) and Elmex (AMF) for remineralization was −8.8% to +6.5%, and the 95% confidence interval of the difference for lesion depth reduction was −6.8% to +4.1%. Therefore, in this study the HAP toothpaste is confirmed to be non-inferior to the fluoride toothpaste in effectiveness.

**Table 1:** Mean rates of remineralization (%) and lesion depth reduction (%) for each toothpaste.

Measurement	Karex	Elmex	*p*-value, two means
% Remineralization	55.8 (s.d. 13.8)	56.9 (s.d. 14.9)	0.81
* p*-value, One group:	<0.0001	<0.0001	
% Lesion depth reduction	27.1 (s.d. 10.6)	28.4 (s.d. 9.8)	0.68
* p*-value, One group:	<0.0001	<0.0001

**Table 2:** Mean (Standard deviation) values of mineral loss (vol%µm) and lesion depth (µm) in the two study groups before and after treatment and their differences (with confidence intervals).

Treatment	Before treatment	After treatment	Difference (95% CI)	*P* value^a^
	ΔZ [Mean (SD)]			
Karex (10%HAP)	2357.5 (454.63)	1013.5 (273.59)	1344 (1119.93–1568.06)	<0.0001
Elmex (500 ppm)	2378.5 (593.16)	1009 (392.48)	1369.5 (1117.40–1621.59)	<0.0001
	LD [Mean (SD)]			
Karex (10%HAP)	92.89 (17.15)	67.07 (11.79)	25.82 (19.79–31.84)	<0.0001
Elmex (500 ppm)	91.91 (17.94)	65.46 (13.64)	26.44 (21.15–31.74)	<0.0001

^a^Paired *t*-test (*n* = 30, *α* = 0.05)

**Fig. 2:**
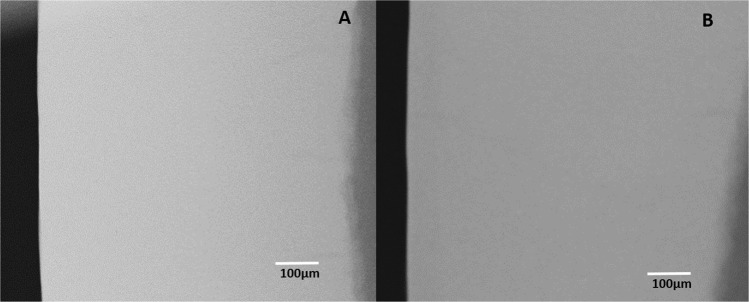
Representative microradiographic images of sound tooth tissue before (**a**) and after (**b**) intra-oral exposure for demineralization while the research subject is using Karex Kid’s toothpaste (10% HAP microclusters).

**Fig. 3:**
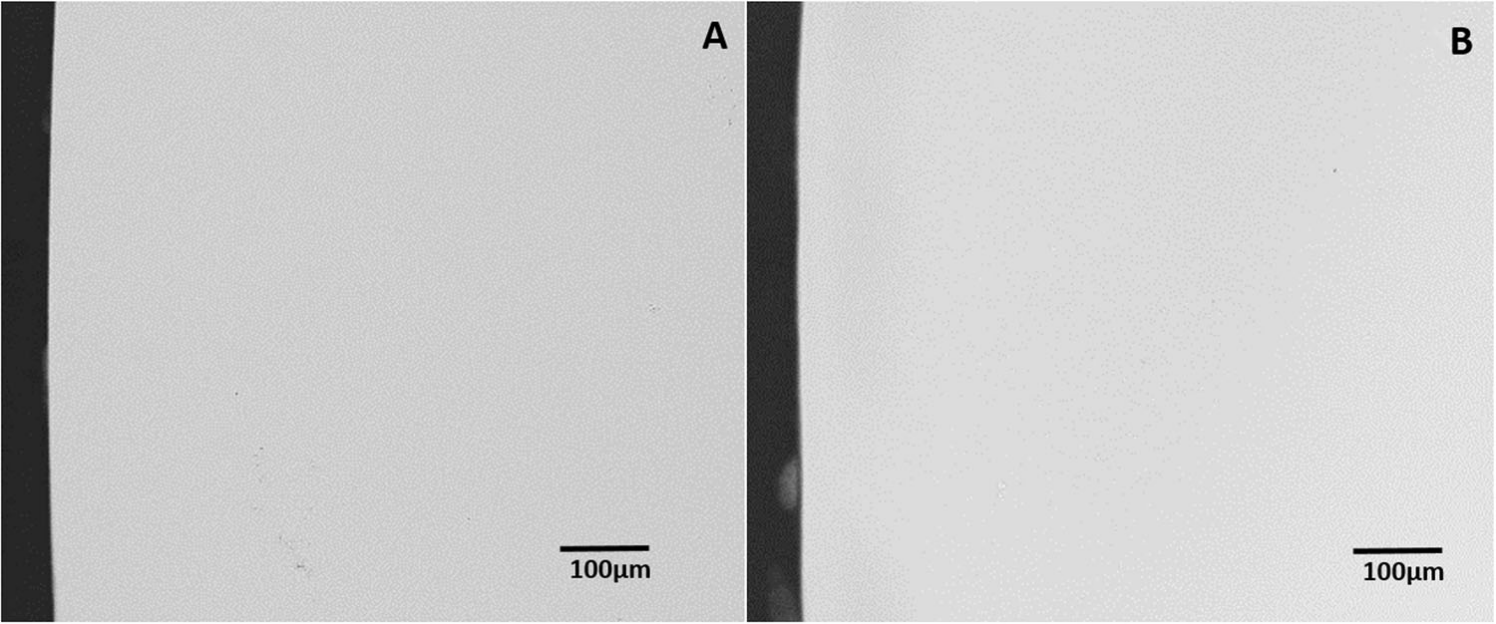
Representative microradiographic images of sound tooth tissue before (**a**) and after (**b**) intra-oral exposure for demineralization while the research subject is using Elmex Kid’s toothpaste (500 ppm fluoride as AMF).

**Fig. 4:**
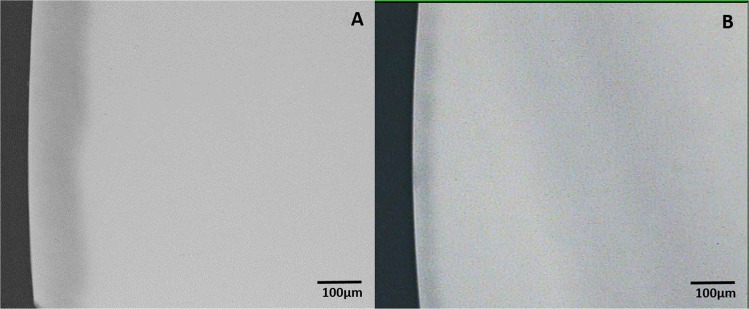
Representative microradiographic images of enamel subsurface lesions (Initial caries lesions), before (**a**) and after (**b**) in situ remineralization via treatment with Karex Kid’s toothpaste (10% HAP microclusters).

**Fig. 5:**
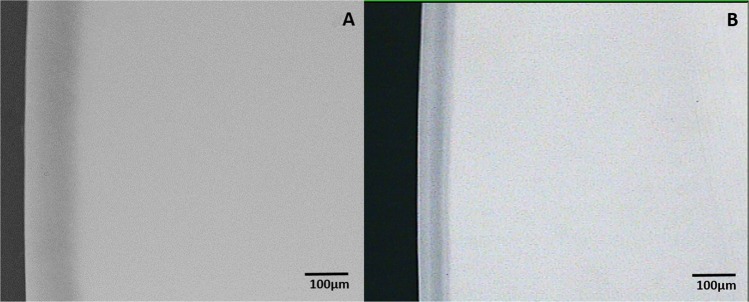
Representative microradiographic images of enamel subsurface lesions (Initial caries lesions), before (**a**) and after (**b**) in situ remineralization via treatment with Elmex Kid’s toothpaste (500 ppm fluoride as AMF).

On analysis of the sound tooth blocks that examined the ability of the two toothpastes to inhibit demineralization of sound tooth surface, there was no evidence of demineralization in any of the tooth blocks following intra-oral exposure to either toothpaste (Fig. 2a, b) and (Fig. 3a, b). Critical examination of the microradiograms from the lesion-bearing samples exposed to HAP (Fig. 4a, b) and AMF (Fig. 5a, b) toothpastes in comparison with their respective control microradiograms, shows that while HAP induced a more homogenous remineralization distributed throughout the entire thickness of the subsurface lesion (Fig. 4b), the remineralization induced by AMF was denser in the out half (surface zone) of the lesion (lesion surface lamination) i.e., two zones of contrasting density can clearly be observed (Fig. 5b). These remineralization patterns in Fig. 4b and 5b were consistent in all specimens exposed to HAP and AMF respectively. There were no incidences of adverse effects reported by subjects or ascertained clinically.

## Discussion

Despite being a preventable disease and amid the presence of fluoride in oral dentifrices, dental caries prevalence in children continues to increase globally,^1^ and at a faster rate among children from low socioeconomic backgrounds.^38^ This indicates that although fluoride interventions have the highest level of supporting evidence as having the most consistent benefit in preventing caries and remineralizing initial caries lesions;^6–8^ additional remineralizing and preventive agents are often needed to enhance fluoride effects in high caries risk individuals.^9,10^ One may suggest increasing the dose of fluoride since its effectiveness is dose-dependent,^7^ unfortunately, there is a limit to fluoride dose allowed in oral care products to avoid the risk of fluorosis in children^17^ and toxicity in all ages.^18,19^ The fluoride dose recommended for toddlers and children is even lower than the regulatory 1000–1500 ppm fluoride concentration in non-prescription toothpastes, which is probably suboptimal for effective remineralization of initial lesions; thus the effect of dose limitation on fluoride effectiveness may be more pronounced in toddlers and children below 6 years. Besides safety concerns, higher fluoride dose in remineralization materials results to surface-zone remineralization at the expense of the lesion body, thus preventing fuller and homogenous remineralization of the lesion.^15,16^ It is envisaged that an agent as effective as fluoride, but can permit increasing dosage for increase effectiveness without safety concerns, may offer a more effective choice, especially for children. Hydroxyapatite, a bioactive and biocompatible material with wide applications in both medicine (e.g., bone substitute) and dentistry, is currently used in nanocrystaline or microcluster forms in toothpaste and mouthrinses in varying concentrations for caries prevention and remineralization.^23–25^ The equivalence (non-inferiority) of 10% HAP in microcluster forms to 500 ppm fluoride provided as AMF in remineralization of initial caries lesion and inhibition of sound enamel demineralization was investigated in this study. The findings of the present in situ study accepted the two hypotheses that each of the two toothpaste formulations promotes remineralization and lesion depth reduction that is significantly greater than zero, and that neither toothpaste is inferior to the other with respect to promoting the remineralization and inhibiting the development of initial caries lesions. Although this study was conducted with human primary teeth, similar result was observed in a previous in situ study that used human permanent teeth.^27^ A randomized controlled clinical trial in children and adolescents at high caries risk undergoing orthodontic treatment also reported similar results and non-inferiority of microcrystalline HAP to 1400 ppm fluoride provided as AMF and stannous fluoride.^32^ Effectiveness of AMF in these reports and this study is in agreement with the long-established fact that fluoride in varying concentrations are effective in preventing caries development and remineralizing initial caries,^9,39,40^ and that the various fluoride salts were equally effective.^41^ Furthermore, Hellwig et al.^42^ in an in situ study demonstrated the remineralization of initial caries lesions of permanent teeth, and concluded that remineralization of primary teeth with fluorides may be possible in the same way as the permanent teeth. The effectiveness of the HAP toothpaste in this study is in agreement with previous clinical and in vitro studies, and it is not surprising considering the various mechanisms through which HAP has been demonstrated to effect remineralization of initial caries.^25,27,28,32,43–47^ Based on its strong affinity and adsorption to tooth surfaces,^24,26,43^ HAP has been shown to induce remineralization of initial caries lesions by directly filling micropores in demineralized tooth surfaces,^26,48^ where it acts as a crystal nucleus, and promotes crystal deposition and growth by continuously attracting large amounts of calcium and phosphate ions from the surrounding remineralization solution.^48,49^

The absence of any evidence of demineralization in all the sound tooth blocks following intra-oral exposure to either HAP or AMF toothpaste further demonstrated the inhibition of demineralization by both toothpastes. The caries prevention potential of HAP, which has been established in previous studies,^31,32^ has been shown to be based on multiple mechanisms. HAP in toothpaste has been reported to elevate calcium and phosphate ions concentrations in saliva, plaque and tooth surfaces;^50^ thus acting as a calcium and phosphate reservoir, helping to maintain a topical state of supersaturation of these ions with respect to tooth minerals.^43,51^ The discussed mechanism must have applied in this study considering that the surface of the tooth blocks was covered with polyesther gauze, which encouraged and maintained plaque accumulation over the tooth surface, providing a nest for the accumulations of the mineral ions. The high potential of HAP to adsorb to bacterial cell wall has been shown to facilitate an antibiofilm effect by inducing coaggregation of bacteria within the HAP particles,^46^ thus aiding biofilm removal from the tooth surfaces,^52,53^ and hindering oral biofilm formation.^29,46,47,54^ Again, this mechanism may have contributed the present findings by limiting the virulence of the biofilm on the surface of the tooth blocks.

The findings in this study further confirmed the surface zone remineralization by the fluoride agents.^13,14,55^ Two zones of contrasting density can clearly be seen in Fig. 5b, showing the remineralization induced by AMF to be denser in the out half of the lesion (surface zone). Observation of such surface zone remineralization with only 500 ppm fluoride present in the toothpaste used in this study actually demonstrated that this ‘lesion lamination’ effect is not limited to materials with high fluoride concentration, rather it is based on the established fact that fluoride remineralization of initial lesions, irrespective of the concentration, is most effective at the outer 30 μm of the lesion.^13,14,55^ This is supported by the findings of a previous study, which reported that higher fluoride concentrations did not produce any further significant increase in remineralization, rather laminations (surface zone remineralization) were apparent in lesions subjected to the 250-ppm and 500-ppm F^−^ solutions.^55^ Another in situ study demonstrated no significant difference in the effectiveness of 500, 1000, and 1500 ppm fluoride in remineralizing initial caries in primary teeth,^42^ further confirming the effect of lesion lamination. The findings of these previous studies and the lesion lamination effect demonstrate that the dose-dependent effect of fluoride effectiveness that reflects as increased effect of high fluoride toothpaste^41^ or addition of further fluoride sources,^56^ has limit at which it plateaus and further increase may not improve the effectiveness. In contrast to the effect of AMF, Fig. 4b shows that HAP induced a more homogenous remineralization distributed throughout the entire thickness of the subsurface lesion, and this may indicate that increasing the dose of HAP or continued usage of the toothpaste may result to increased remineralization of the lesion, and ultimately lead to complete or fuller remineralization of the initial lesion.

Based on above discussions one may suggest that HAP-containing toothpaste may be a better choice for children and individuals at high caries risk since the dosage can be increased to obtain higher efficacy without any safety issue such as the risk of fluorosis in children associated with high fluoride dose. Furthermore, the use of HAP in oral care products may eliminate the need of combining fluoride and antimicrobials in a dentifrice, as well as having different dosages for infants, children and adults. It is logical but scientific that since the remineralizing efficacy of topical fluorides is strictly dependent on the availability of calcium and phosphate ions, HAP dentifrices may be a more effective for xerostomic patients with diminished amounts of saliva. This may need to be confirm through a clinical trial on patients suffering from xerostomia.

In this study, there was no incidences of adverse effects reported by subjects or ascertained clinically. Previous clinical studies observed similar findings, and reported there is no safety issue with HAP in oral care products.^27,32,57^ The fact that these studies investigated varying doses of HAP in toothpastes, and none reported any safety issue, confirms that the dose of HAP in oral care dentifrices can be safely increased for an increase effectiveness when necessary.

## Conclusion

- This study confirmed hydroxyapatite toothpaste is equivalent or non-inferior to the fluoride toothpaste with respect to remineralization of initial caries lesions and prevention of carious lesion development.
- Future research should be large multicenter clinical trials to further establish the effectiveness of HAP dentifrices and its equivalence to fluoride.
